# Impact Evaluation of the Kenya Frontline Field Epidemiology Training Program: Repeated-Measures Study

**DOI:** 10.2196/18956

**Published:** 2021-01-08

**Authors:** Zeinab Gura Roka, Elvis Omondi Oyugi, Jane Njoki Githuku, Evalyne Kanyina, Mark Obonyo, Victor Omballa, Waqo Gufu Boru, James Ransom

**Affiliations:** 1 Ministry of Health Field Epidemiology and Laboratory Training Program Nairobi Kenya; 2 Ministry of Health National Malaria Program Nairobi Kenya; 3 Ministry of Health Emergency Operations Centre Directorate of Public Health Nairobi Kenya; 4 Food and Agriculture Organization United Nations Subregional Office of Southern Africa Harare Zimbabwe; 5 Bioreference Laboratories Elmwood Park, NJ United States; 6 Piret Partners Consulting Washington, DC United States

**Keywords:** field epidemiology, workforce development, Kenya, training

## Abstract

**Background:**

In 2014, Kenya’s Field Epidemiology and Laboratory Training Program (FELTP) initiated a 3-month field-based frontline training, Field Epidemiology Training Program (FETP-F), for local public health workers.

**Objective:**

This study aimed to measure the effect of FETP-F on participant workplace practices regarding quality and consistency of public health data, critical interaction with public health data, and improvements in on-time reporting (OTR).

**Methods:**

Between February and April 2017, FELTP conducted a mixed methods evaluation via online survey to examine outcomes achieved among all 215 graduates from 2014 and 2015. Data quality assessment (DQA) and data consistency assessment (DCA) scores, OTR percentages, and ratings of the training experience were the quantitative measures tracked from baseline and then at 6-month intervals up to 18 months postcompletion of the training. The qualitative component consisted of semistructured face-to-face interviews and observations. Quantitative data were analyzed using descriptive statistics and one-way analysis of variance (ANOVA). Qualitative data were transcribed and analyzed to identify key themes and dimensions.

**Results:**

In total, 103 (47%) graduates responded to the survey. Quantitative analyses showed that the training significantly increased the mean DQA and OTR scores but there was a nonsignificant increase in mean DCA scores. Qualitative analyses found that 68% of respondents acquired new skills, 83% applied those skills to their day-to-day work, and 91% improved work methods.

**Conclusions:**

FETP-F improved overall data quality and OTR at the agency level but had minimal impact on data consistency between local, county, and national public health agencies. Participants reported that they acquired practical skills that improved data collation and analysis and OTR.

## Introduction

Strengthened health systems played a key role in improving global life expectancy throughout the 20th century [1]. For the 21st century, public health workforce competencies have important implications for global health preparedness, local disease surveillance and response capacity, health systems infrastructure, and overall population health outcomes [2].

The Field Epidemiology Training Program – Frontline (FETP-F) is a 3-month competency-based, service-oriented collaborative training program that is anchored within the Kenya Ministry of Health (MoH) [3]. The partners of FETP-F include the Ministry of Agriculture, Livestock and Fisheries; the Centers for Disease Control and Prevention (CDC), Kenya Medical Research Institute (KEMRI), and county and subcounty health departments and hospitals [4].

The first phase of frontline training was implemented between September 2014 and December 2016 throughout all 47 counties in Kenya, with a goal of improving local frontline health workers’ ability to detect, report, and respond to unusual health events [5].

## Methods

Between February and April 2017, the Field Epidemiology and Laboratory Training Program (FELTP) used quantitative, semiquantitative, and qualitative methods to evaluate all FETP-F activities. A survey link was sent to all 215 graduates of Groups 1-6 because they graduated >18 months before the impact evaluation began.

### Quantitative Measures

We used interrupted repeated measures on 3 quantitative values (data quality assessment [DQA], data consistency assessment [DCA], and on-time reporting [OTR]) at 6, 12, and 18 months postgraduation from FETP-F. For all quantitative measures, one-way analysis of variance (ANOVA) analysis was performed using Microsoft Excel’s (Microsoft Corp) Data ToolPak.

#### DQA Scores

The participants completed a DQA for their field project, and we used those scores as baseline. The DQA tool was designed for the following tasks: (1) verify the quality of health facility data, (2) assess the system that produces that data, and (3) develop action plans to improve items 1 and 2.

#### DCA Scores

The DCA is an end-to-end data integrity process that focuses on the entire surveillance network. The first end is the generation of data at the health facility level. The middle is the county record, where the health facilities report their weekly and monthly tallies. The last end is when data are entered into the District Health Information System (DHIS) by the county Health Records and Information Officer (HRIO). The goal is to detect inconsistencies as data travel through the surveillance system and identify root causes for these inconsistencies.

#### Timeliness of Reporting

Timeliness is a key performance measure of public health surveillance systems. We used the results from the field project as baseline OTR measures, and then followed up at 6, 12, and 18 months postgraduation.

### Semiquantitative Measures

At the beginning of each training course, we asked participants to score their knowledge and skills in 8 key competencies on a Likert scale from 1 to 5, with 1 representing limited knowledge/skills and 5 representing expertise. We used those scores to gauge the impact of FETP-F training on knowledge, skills, and change in work methods.

Semistructured interviews were conducted with randomly selected graduates from groups 1-6, because we wanted to examine the impact of the training at least 1.5 years postgraduation; this meant that we could only look at the impact of FETP-F on the work methods of the first 6 groups to complete the FETP-F process.

### Ethics Approval and Consent to Participate

Informed consent was obtained from all FETP-F graduates who agreed to an evaluation visit. Personal identifiers were not included in the recorded data. Permission to conduct this evaluation was sought from and granted by the Ethical Review Board of the Ministry of Health (FAN: IREC 1795). This evaluation did not involve any animal subjects. The evaluation did not collect human subject data nor any human specimen samples. All subjects provided signed and oral consent for participation. Informed consent included consent to publish findings of this evaluation research. This research did not use any images, names, or other identifying information of any of those who consented for interview and participation in the evaluation. Therefore, a consent for publication was not needed from any of the research subjects.

## Results

### Demographics of Survey Respondents

Overall, 103 graduates representing all regions of the country were included in the analyses. Most (55%) were male and 60% (n=62) had <10 years of public health work experience. The breakdown by cadre was the following: 20% (n=21) medical officers, 15% (n=15) veterinary officers, 25% (n=26) public health officers, 15% (n=15) laboratory staff, 15% (n=16) nursing staff, and 10% (n=10) other.

### DQA Scores

Descriptive analyses of 103 DQA scores from baseline to 18 months postgraduation showed an increase in the mean DQA score from 75.6% at baseline to 84.5% at 18 months postgraduation.

Table 1 shows a 10.5% improvement in the mean DQA score for this sample of health facilities and programs. The subsequent ANOVA analyses on the 103 respondents showed that although the improvement was only 10.5%, this represented a significant improvement in DQA mean scores since baseline.

**Table 1:** Repeated-measures scores for data quality assessment, data consistency assessment, and on-time reporting of Kenya Field Epidemiology Training Program–Frontline graduates, 2014-2015.

Results and time interval postgraduation		Mean (SD)
**Analysis of variance results, data quality assessment mean scores^a^**		
	Baseline	75.64 (8.05)
	6 months	74.88 (9.00)
	12 months	75.08 (5.21)
	18 months	84.53 (8.82)
**Analysis of variance results, data consistency assessment mean scores^b^**		
	Baseline	73.22 (27.59)
	6 months	68.11 (13.42)
	12 months	78.22 (21.46)
	18 months	82.66 (21.37)
**Analysis of variance results, on-time reporting mean scores^c^**		
	Baseline	29.66 (15.58)
	6 months	70.11 (23.39)
	12 months	70.83 (180.1471)
	18 months	74.88 (624.3399)

^a^Between-groups: *F*=70.71; f-crit=2.61; *P*<.001.

^b^Between-groups: *F*=0.765; f-crit=2.90; *P*=.52.

^c^Between-groups: *F*=20.37, f-crit=2.74, *P*<.001.

### DCA Scores

Descriptive analyses of DCA scores showed that there was an 11.4% improvement in DCA scores between baseline and 18 months postgraduation. However, upon further analyses using ANOVA, results showed that the increase was not significant (Table 1).

### OTR Proportions

We examined the proportion of monthly reports submitted on time from health facilities to county health departments for the preceding quarter (Table 1). Analyses show that there was a >60% increase in OTR between baseline and the 18-month assessment. The ANOVA showed this to be a significant development and improvement compared to baseline values.

### Semiquantitative Self-assessment of Learning Scores

Knowledge/skill levels for the 8 assessed competencies were relatively low before the training. After training, we noted significant increases in the mean knowledge/skill scores in each of the 8 competencies. During the site visits, field workers also interviewed supervisors of the graduates and at least one colleague regarding any notable changes (positive or negative) after the graduate resumed his/her normal work duties. We used the same assessment scale as with the graduates. Comparisons of mean difference scores among FETP-F graduates, their supervisors, and their colleagues in 8 competency areas are outlined in Table 2, using a Likert scale between 1 and 5.

There was not much variation in the self-assessments of the graduates when compared to the assessments of competencies provided by their supervisors and colleagues. However, the supervisors and colleagues noted a marked increase in Microsoft Excel skills, knowledge, and expertise postgraduation.

For the larger group of graduates (n=103), we examined via online survey the mean skills and knowledge changes (pre-post) in the key competencies before training (pretraining), immediately after the 3-month session ended (posttraining), and 18 months after training (follow-up; Table 3).

**Table 2:** Learner, supervisor, and colleague assessment of pre-post scoring of learner knowledge and skill in key competencies.^a^

Competency	Self-score (n=19)	Supervisor score (n=12)	Colleague score (n=7)
Statistics	1	2	2
Epidemiology	3	2	3
Surveillance	2	2	2
Microsoft Excel	1	3	2
Data analysis	2	1	2
Field investigations	2	2	2
Data audits	3	3	3
Communicating public health data	2	2	Not reported

^a^The assessment scale ranged from 1 to 5 (1=no skills, 2=limited skills, 3=average skills, 4=good skills, and 5=mastery). Classification of the difference scores tabulated above are in terms of improvement: 0=none, 1=limited , 3=modest, and >3=significant.

**Table 3:** Changes in knowledge and skills of Field Epidemiology Training Program–Frontline graduates, 2014-2015 (n=103)^a^.

Competency	Time of measurement		
	Pretraining, mean (SD)	Posttraining, mean (SD)	Follow-up, mean (SD)
Statistics	2.77 (0.81)	3.69 (0.61)	4.35 (0.72)
Epidemiology	2.68 (0.72)	4.11 (0.45)	3.74 (0.69)
Surveillance	2.82 (0.73)	3.84 (0.59)	3.99 (0.51)
Microsoft Excel	1.86 (0.75)	3.81 (0.55)	3.97 (0.62)
Data analysis	2.55 (0.96)	3.95 (0.69)	3.56 (0.49)
Field investigations	2.32 (0.89)	3.47 (0.82)	2.66 (0.74)
Data audits	2.86 (0.99)	3.89 (0.55)	3.82 (0.62)
Communicating public health data	2.73 (0.58)	3.94 (0.31)	4.02 (0.47)

^a^The ordinal scale ranged from 1 to 5 (1=no knowledge, 2=little knowledge, 3=average, 4=good, and 5=mastery). Pretraining occurred before the training, posttraining occurred immediately after completing the 3-month training process, and follow-up was performed at least 18 months postgraduation from the training program. Between-groups: *F*=30.02; f-crit=3.47; *P*<.001.

### Qualitative Results

Field investigators visited 19 sites and conducted 38 one-on-one private interviews (with graduates, supervisors, and colleagues). We analyzed the transcripts of all interviews (n=19 graduates, n=12 supervisors, and n=7 colleagues). After transcription, we conducted 3 levels of analysis. The coding process was iterative and involved multiple stages that involved preparing and formatting the raw data so that they are available for evaluation.

After conducting the first-level analyses using keyword searches and generating word clouds, we had a list of 107 codes. During the second-level analyses, we reduced the codes from 107 to 37, which we later grouped into 25 themes. After the third-level review, we noted that the themes clustered into 3 key dimensions. Graduates, their supervisors, and their colleagues’ comments were associated with “personal” aspects (benefits to self), organizational aspects (benefits to the agency or organization where the graduate worked or health partners in the graduate’s community), and the FETP process itself (feelings and perspectives on the nominations/selection process, the execution of the course inclusive of its contents, and feedback on the quality of the faculty and facilitators) [6].

## Discussion

### Principal Findings

Field epidemiology training programs worldwide are based on multiple administrative models. Our evaluation results show the effectiveness of a localized field epidemiology and data management training process for improving the skills and capacity of frontline health workers. During the interviews, most graduates, their supervisors, and their colleagues reported that the course had helped them to make scientifically based decisions and improved their overall capacity to deal with a spectrum of public health challenges, from calculating thresholds to responding to cholera cases. Additionally, they reported that the course helped them to become better leaders by improving their communication skills, enabling them to make more evidence-based decisions, and empowering them to show colleagues how to practically interact more critically with the data they generate at their agencies. Our findings align with evaluation results from other FETPs. In both Japan and Mongolia, the positive effect this approach had on trainees was demonstrated in post–training of trainers evaluations and posttraining application of knowledge and skills [7,8].

Several other examples have clearly showed the success of FETP in responding to emergencies and disasters [9]. During the Middle East Respiratory Syndrome (MERS) outbreak in 2014 in Saudi Arabia, FETP graduates tackled numerous issues, including redesigning the system to enable simultaneous real-time electronic reporting of suspected and confirmed cases to public health professionals who needed to take essential control and preventive actions on new cases [10]. FETPs in the Eastern Mediterranean Region showed success in building the epidemiologic capacity of the public health workforce, improving countries’ surveillance systems, and strengthening health systems [9].

One of the strengths of this study is that we assessed “the degree of applying what was learned” and “the degree to which outcomes occur as a result of the training,” which are levels 3 and 4 of the Kirkpatrick model, respectively [10]. Another strength is that the evaluation was based on information from two sources, including the FETP graduates and program advisers, who are within the health system at a level where they can observe the impact of the program.

Our results were derived from an online survey, with all the potential strengths and limitations of that medium. The survey was anonymous and, thus, it is very likely that participants gave accurate answers without fear of exposing their identity. In addition, they were not under any pressure to give “desirable answers” to the survey questions. Although the response rate was only about 55%, this is more than expected with this type of survey.

Implementing this approach revealed some challenges: first, the approach requires assessment of participant learning needs and subsequent systematic training design; thus, facilitators must review and redesign curricula for each event. Second, participatory methods can be new and uncomfortable for individuals educated in formal or traditional styles, implying that programs with longer records and institutional memory may be hesitant to change. Third, systematically evaluating short- and long-term effects of this approach beyond pretest and posttest questionnaires was challenging; therefore, program administrators should develop careful impact evaluations that begin before training. Finally, the approach requires a facilitator who is skilled and comfortable with participatory methods.

Some additional limitations of the current evaluation should be noted. First, the bulk of the data collected are self-reported, including DQA, DCA, and OTR scores, as well as measurements of respondents’ perceptions of learning and impact. It is possible that participants overrated or underrated their skills and knowledge when responding to survey items online. Second, the time gap from delivery of the course to data collection could have affected the information that graduates gave to us. Additionally, the data collection had to be rushed due to pending funding cuts. This will hinder subanalyses of the formative and summative evaluation data over the life of the project. Further efforts are needed to determine if skills and/or benefits from the course change over time and whether the documented improvements in health facility data quality, consistency, and OTR change over time, particularly as replications continue and the time gap since training widens and we lack a steady flow of their colleagues who can participate in such training.

Further, many of the graduates did not respond to the repeated-measures surveys, so we do not have relevant data about them; therefore, we are not able to conclude that respondents are a representative sample of graduates.

Finally, we know that FETP-F’s participants take part in a never-ending array of trainings, so we do not know how those other trainings have impacted the findings that we have documented. In addition, we do not know the spectrum of participants’ involvement in support networks, how the doctors’ and nurses’ strikes affected outcomes, the role of politics in who is nominated to participate in the training, local rates of job turnover, and if there is an effect on uptake among some younger public health workers associated with the fact that the FETP-F does not award a diploma.

In summary, FETPs that plan to build sustainable public health response capacity and expertise from its most local levels for handling public health threats across health sectors should consider incorporating this approach, which combines participatory methods and periodic follow-up assessments with retraining opportunities and concurrent impact evaluations. This will improve governments’ understanding of their public health workforces’ potential for improving capacity to meet global epidemiology goals [5].

### Conclusions

FETP-F is a viable and effective method for improving Kenya’s public health workforce’s skills, knowledge, and practices in key competencies. This evaluation suggests many benefits and lessons on frontline field epidemiology training including the following: (1) the advantage of focusing on local health workers who are more familiar with contextual issues to allow tailoring of the training, (2) enhanced collaboration among multiple practice cadres to create a forum for networking and new partnership opportunities, (3) a more convenient method of training that eliminates the need to bring in external trainers or for participants to travel outside of their region, and (4) specific examples of how to improve future iterations of this kind of training. This evaluation suggests that the FETP-F model has increased the capacity of local health workers trained in field epidemiology and data analytics, while maintaining fidelity with the original objectives and frameworks of the original model, the advanced-level field epidemiology training program. The FETP-F met its aims and objectives satisfactorily, and resulted in positive shifts in knowledge, attitudes, and behavioral intentions of local health workers who graduated from the program. This suggests that this training strategy was effective and feasible in improving the capacity of local public health workers of all cadres.

## Abbreviations

ANOVA: analysis of variance
CDC: Centers for Disease Control and Prevention
DQA: data quality assessment
DCA: data consistency assessment
DHIS: District Health Information System
FELTP: Field Epidemiology and Laboratory Training Program
FETP: Field Epidemiology Training Program
HRIO: Health Records and Information Officer
KEMRI: Kenya Medical Research Institute
MoH: Ministry of Health
OTR: on-time reporting
